# The PerioGene North Study Uncovers Serum Proteins Related to Periodontitis

**DOI:** 10.1177/00220345241263320

**Published:** 2024-08-05

**Authors:** M. Wänman, S. Betnér, A. Esberg, C.K. Holm, C. Isehed, A. Holmlund, P. Palmqvist, A. Lövgren, S. Lindquist, L. Hänström, U.H. Lerner, E. Kindstedt, P. Lundberg

**Affiliations:** 1 Department of Odontology, Umeå University, Section for Molecular Periodontology, Umeå, Sweden; 2 Northern Registry Centre, Department of Public Health and Clinical Medicine, Umeå University, Umeå, Sweden; 3 Department of Odontology, Umeå University, Umeå, Sweden; 4 Gävle County Hospital, Department of Periodontology, Public Dental Health County Council of Gävleborg, Gävle, Sweden; 5 Center for Research and Development Uppsala University/Region Gävleborg, Gävle, Sweden; 6 Department of Periodontology, County Council of Västerbotten, Umeå, Sweden; 7 Department of Odontology, Umeå University, Section for Clinical Oral Physiology, Umeå, Sweden; 8 Lipum AB, Umeå, Sweden; 9 Sahlgrenska Ostoporosis Centre, Centre for Bone and Arthritis Research, Department of Internal Medicine and Clinical Nutrition, Institute for Medicine, Sahlgrenska Academy at University of Gothenburg, Gothenburg, Sweden

**Keywords:** proteomics, inflammation, bone loss, periodontal disease, epidemiology, biomarkers

## Abstract

The sequalae of periodontitis include irreversible degradation of tooth-supporting structures and circulatory spread of inflammatory mediators. However, the serum protein profile in periodontitis is not well described, which is partly attributable to the limited number of studies based on large and well-characterized periodontitis cohorts. This study aims to identify novel, circulating inflammation-related proteins associated with periodontitis within the PerioGene North case-control study, which includes 478 cases with severe periodontitis and 509 periodontally healthy controls. The serum concentrations of high-sensitivity C-reactive protein (hs-CRP) and a panel of 45 inflammation-related proteins were analyzed using targeted proteomics. A distinguishable serum protein profile was evident in periodontitis cases. The protein pattern could separate cases from controls with a sensitivity of 0.81 and specificity of 0.81 (area under the curve = 0.87). Adjusted levels for hs-CRP and 24 of the 45 proteins were different between cases and controls. High levels of hs-CRP and matrix metalloproteinase–12, and low levels of epidermal growth factor (EGF) and oxidized low-density lipoprotein receptor 1 (OLR-1) were detected among the cases. Furthermore, the levels of C-C motif chemokine–19, granulocyte colony-stimulating factor–3 (CSF-3), interleukin-7 (IL-7), and hs-CRP were significantly higher in cases with a high degree of gingival inflammation. The levels of CSF-3 and tumor necrosis factor ligand superfamily member–10 TNFSF-10 were higher in cases with many deep periodontal pockets. The PerioGene North study includes detailed clinical periodontal data and uncovers a distinct serum protein profile in periodontitis. The findings of lower EGF and OLR-1 among the cases are highlighted, as this has not been presented before. The role of EGF and OLR-1 in periodontitis pathogenesis and as possible future biomarkers should be further explored.

## Introduction

Periodontal inflammation is, in most cases, initiated by dysbiotic microbial communities. In periodontitis-susceptible individuals, inflammation triggers innate and adaptive immune responses that cause irreversible degradation of tooth-supporting structures. The destruction of connective and bone tissue is not simply a hallmark of periodontitis but also generates a nutritionally favorable environment that further propagates the bacterial dysbiosis through a negative pathogenic loop (Hajishengallis et al. 2020).

In periodontitis, tissue resident and infiltrating immune cells collectively create an imbalance in the ratio between pro- and anti-inflammatory cytokines, activating pathways that lead to tissue destruction (Yucel-Lindberg and Bage 2013). Increased levels of tumor necrosis factor (TNF)–α and interleukin (IL)–1β, –6, and –17 trigger the expression of, for example, proteolytic matrix metalloproteinases (MMPs) and osteoclastogenic receptor activator of nuclear factor kappa-B ligand (RANKL) (Souza and Lerner 2013). Besides insufficient resolution of inflammation, evidence suggests that periodontitis is associated with impaired wound healing. Growth factors play a major role during healthy periodontal tissue turnover by modulating healing processes in a coordinated manner (Cho et al. 2021). Furthermore, these molecules affect periodontal repair and regeneration during chronic inflammatory conditions. Most prominent among these cell-derived members are the epidermal growth factor (EGF) family and transforming growth factor beta (Kaigler et al. 2006).

Increasing evidence pinpoints a systemic spread of bacteria, microbial products, and inflammatory mediators through the damaged epithelium that delineates the periodontal pocket. Previous reports indicate higher levels of acute phase proteins and proinflammatory cytokines in serum from individuals with periodontitis (Forner et al. 2006; Paraskevas et al. 2008; Bostrom et al. 2015; Pussinen et al. 2022). Low-grade systemic inflammation is a probable mechanistic link between periodontitis and related comorbidities, for example, diabetes mellitus, Alzheimer’s disease, and cardiovascular disease (CVD) (Hajishengallis 2015). However, most studies that have investigated serum proteins in periodontitis are based on small, inadequately characterized cohorts with limited information about risk factors.

As inflammatory and immunologic disease processes have an impact on the composition of circulating body fluids, changes in protein levels in blood can be used as biomarkers for disease recognition (Han et al. 2020). In addition, the definition of serum protein profiles can reveal key pathological mechanisms of a disease (Kondo et al. 2021). Thus, candidate drug targets have been based on serum/plasma protein analyses in different diseases (Monaco et al. 2015).

The multifactorial nature of periodontitis is a challenge when trying to identify serum proteins associated with the disease. However, large well-characterized periodontitis cohorts could overcome this obstacle and uncover molecules with a hitherto unknown role in periodontitis pathogenesis and importance for development of comorbidity.

In this study, we aim to extend the current knowledge on the serum protein profile in severe periodontitis within the large and well-characterized PerioGene North study.

## Materials and Methods

### Ethical Statement

This study was approved by the Regional Ethical Review Board at Umeå University and Uppsala University. Amendments were obtained by the Swedish Ethical Review Authority. Furthermore, the study was performed in accordance with the Declaration of Helsinki and conformed to the Strengthening the Reporting of Observational Studies in Epidemiology (STROBE) guidelines.

### Study Design

PerioGene North is a multicenter case-control study consisting of 526 periodontitis cases and 532 periodontally healthy controls. The study participants were consecutively recruited between 2007 and 2019, from specialist clinics and general dental care within the counties of Västerbotten, Gävleborg, Uppsala, and Västmanland in northern Sweden. The cases were examined by senior consultants in periodontology and the controls by general dentists. To validate the absence of alveolar bone loss in controls, all radiographs were reviewed by senior consultants in periodontology.

### Clinical Data Collection

All participants underwent a complete oral and periodontal examination, including registration of bleeding on probing (BoP) and periodontal probing pocket depth (PPD), at 6 sites per tooth using a PCP-12, 3-6-9-12 (Hu-Friedy) dental probe. Furcation involvement was assessed but not registered in the study protocol. Alveolar bone loss was assessed for each tooth using dental radiographs (bitewing and apical images). Information regarding number of teeth per quadrant with PPD (<4 mm, 4 to 6 mm, and >6 mm) and degree of alveolar bone loss (<1/3, ≥1/3 to ≤2/3 or >2/3 of the root length) was registered in the study protocol. All included cases fulfilled the stage III criteria, and 87 cases (18.2%) with <20 teeth could be classified as stage IV (Holtfreter et al. 2024). However, we cannot confirm that these individuals lost teeth due to periodontitis. We concluded that all cases present with severe periodontitis, meaning stage III to IV according to the currently used classification system (Tonetti et al. 2018b).

The cases were subcategorized based on BoP (high ≥20%, low <20%), PPD, and alveolar bone loss according to previous studies (Holmlund et al. 2011; see appendix). Information about sex, birth country, past and/or current tobacco use, education level, and awareness of parent with periodontitis (self-reported heredity) was recorded. Clinical variables such as height and weight were obtained. Information about general health (diseases according to ICD-10 categories) was obtained from the registries of the National Board of Health and Welfare.

### Inclusion and Exclusion Criteria

The inclusion criteria for cases were (1) having at least 1 tooth in each quadrant with alveolar bone loss ≥1/3 of the root length and (2) having ≥15 remaining teeth or ≥8 if teeth were present only in 1 jaw. Cases with alveolar bone loss that could be explained by local aggravating factors such as root fractures or pulpal infections were excluded. The inclusion criteria for the control group were (1) no alveolar bone loss (i.e., <3-mm distance from the cementoenamel junction to the bone crest), (2) PPD <4 mm, (3) having ≥24 remaining teeth, and (4) being ≥34 y of age. Participants with known contagious blood diseases were excluded.

### Blood Sampling

A nonfasted venous blood sample of 3 × 10 mL was collected at inclusion. Collection and handling of blood samples, including fractionation into plasma, serum, and buffy coat, and storage at −80 °C followed the standardized routines at Medical Biobank of Northern Sweden, Västerbotten County Council, Sweden.

### Serum Analysis

A panel of inflammatory-related proteins was analyzed with a DNA-based proximity extension assay (Olink Target 48 Cytokine Panel; see Appendix Table 1), which includes 45 proteins and 3 internal controls. High-sensitivity C-reactive protein (hs-CRP) levels were assessed using a V-PLEX human CRP kit on a Mesoscale platform. All serum analyses were executed by SciLife Lab, Uppsala University (Uppsala, Sweden).

### Statistical Analysis

Descriptive analyses were used for frequency distributions of categorical variables, whereas medians with interquartile range were calculated for continuous variables. Group comparisons were conducted using either chi-square tests for categorical variables or Mann–Whitney *U* test for continuous variables. Missing values of proteins were imputed using a random forest imputation algorithm. A uniform manifold approximation and projection (UMAP) was performed to visualize the difference in protein patterns between cases and controls. A receiver-operating characteristic (ROC) curve was used to evaluate the separation between cases and controls. Youden’s J statistic was used to determine the cutoff with the highest sensitivity and specificity. Protein levels in relation to periodontitis, gingival inflammation, PPD, and alveolar bone loss were assessed using a linear regression model with log-transformed concentrations as the outcome variable. The models were adjusted for the effect of age, sex, and ever smoked. Results were presented as exponential regression coefficient (Exp[B], interpretable as relative effect), 95% confidence interval (CI), and *P* value. Adjustment for multiple testing was performed using the Benjamini and Hochberg method. Due to the skewness in age between cases and controls, post hoc analyses with age-stratified 10-y clusters were performed for hs-CRP, EGF, oxidized low-density lipoprotein receptor 1 (OLR-1), and MMP-12, respectively. A *P* value of <0.05 was considered statistically significant. The Statistical Package for Social Sciences (SPSS) version 26 (IBM Corporation) and R version 4.2.3 (R Foundation for Statistical Computing) were used for the statistical analysis.

## Results

### Characteristics of the Study Participants

A total of 1,058 individuals were enrolled in the PerioGene North study. Here, 60 subjects were excluded due to missing clinical data, and 11 subjects were excluded due to not fulfilling the inclusion criteria. This resulted in a total of 987 study participants, of whom 478 were cases and 509 were controls. Six subjects did not donate blood and were therefore not included in the serum analyses. The characteristics of the study participants are shown in Table 1, and missing values for each variable are reported in Appendix Table 2. The distribution of men and women was equal among the groups; however, the cases were older and had a higher body mass index. The cases also presented with a higher proportion of ever smokers and more frequently reported heredity for periodontitis. The controls had a higher education level as well as higher numbers of previous and current snuff users. There was no significant difference in the prevalence of the general diseases analyzed between cases and controls after adjusting for confounders (see Table 1 and Appendix Table 3).

**Table 1.** Characteristics of the PerioGene North Study Participants.

	Control (*n* = 509)	Case (*n* = 478)	*P* Value^a^
Characteristic			
Male, *n* (%)	218 (42.8)	198 (41.4)	0.699
Female, *n* (%)	291 (57.2)	280 (58.6)	
Age at examination, median (IQR)	44.0 (39.0–50.0)	59.0 (49.0–66.0)	<0.001
Body mass index, median (IQR)	25.2 (23.2–28.0)	26.1 (23.1–29.0)	0.019
Education level, *n* (%)			
Elementary school	21 (4.1)	141 (29.5)	<0.001
High school	117 (23.0)	151 (31.6)	
University	371 (72.9)	185 (38.7)	
Tobacco, *n* (%)			
Ever smoked	105 (20.6)	358 (74.9)	<0.001
Present smoker	13 (2.6)	125 (26.2)	<0.001
Ever snuff	133 (26.1)	82 (17.1)	<0.001
Present snuff user	82 (16.1)	57 (11.9)	<0.001
Parent with periodontitis, *n* (%)			
No	19 (3.7)	6 (1.3)	<0.001
Yes	48 (9.4)	137 (28.7)	
Do not know	442 (86.8)	335 (70.1)	
Country of birth, *n* (%)			
Sweden	491 (96.5)	410 (85.8)	<0.001
Other	18 (3.5)	68 (14.2)	<0.001
Periodontal parameters, median (IQR)			
Number of teeth	28.0 (27.0–28.0)	24.0 (21.0–27.0)	<0.001
BoP %	5.0 (1.0–12.0)	25.0 (13.0–44.0)	<0.001
No. of teeth with PPD < 4mm	25.0 (20.0–28.0)	6.0 (2.0–11.0)	NA
PPD 4 to 6 mm	0.0 (0.0–0.0)	11.0 (7.0–15.0)	NA
PPD >6 mm	0.0 (0.0–0.0)	3.0 (1.0–7.0)	NA
Alveolar bone loss <1/3 of root length	0.0 (0.0–0.0)	10.0 (4.0–15.0)	NA
≥1/3 to ≤2/3	0.0 (0.0–0.0)	8.0 (5.0–12)	NA
>2/3	0.0 (0.0–0.0)	4.0 (2.0–6.0)	NA
Periodontal parameter categorization			
BoP level, *n* (%)			
Low (<20%)	431 (84.7)	185 (38.7)	<0.001
High (≥20%)	78 (15.3)	291 (60.9)	<0.001
PPD level,^b^ *n* (%)			
None	NA	7 (1.5)	NA
Low	NA	75 (15.7)	NA
Moderate	NA	193 (40.4)	NA
High	NA	203 (42.5)	NA
Alveolar bone loss level,^b^ *n* (%)			
Low	NA	117 (24.5)	NA
Moderate	NA	246 (51.5)	NA
High	NA	115 (24.1)	NA
General diseases (ICD-10 code),^c^ *n* (%)			
Cancer (C0-99)	15 (2.9)	31(6.5)	0.748
Diabetes type 1 (E10)	4 (0.8)	10 (2.1)	0.187
Diabetes type 2 (E11)	2 (0.4)	19 (4.0)	0.123
Obesity (E65-66)	6 (1.2)	15 (3.1)	0.121
Cardiovascular disease (I0-99)	47 (9.2)	103 (21.5)	0.970
High blood pressure (I10-15)	21 (4.1)	70 (14.6)	0.397
Ischemic heart disease (I20-25)	3 (0.6)	34 (7.1)	0.114
Cerebrovascular conditions (I60-69)	2 (0.4)	10 (2.1)	0.301
Lung disease (J40-45)	20 (3.9)	22 (4.6)	0.534
Rheumatoid arthritis (M05-06)	2 (0.4)	3 (0.6)	0.604
SLE (M32)	0 (0)	1 (0.2)	NA
Osteoporosis (M80-81)	1 (0.2)	6 (1.3)	0.544
Inflammatory bowel disease (K50-55)	7 (1.4)	8 (1.7)	0.886

BoP, bleeding on probing; IQR, interquartile range; NA, not applicable; PPD, pocket probing depth; SLE, systemic lupus erythematosus.

a *P* value regarding general diseases is based on the logistic regression model with case and control as the dependent variable. Adjusted for the effects of age, gender, and ever smoked.

b Group categorization was based on a PPD/alveolar bone loss score. None = ≤1.00, low = 1.01 to 1.49, moderate = 1.50 to 1.99, high = ≥2.00. The score was calculated by assigning each tooth a score of 1 to 3 depending on the degree of PPD/alveolar bone loss. The total score for the entire dentition was summed and then divided by the number of teeth.

c Cardiovascular disease is a merged category for high blood pressure, ischemic heart disease, and cerebrovascular conditions.

### Individuals with Periodontitis Have a Distinguishable Serum Protein Profile

**Figure 1.**
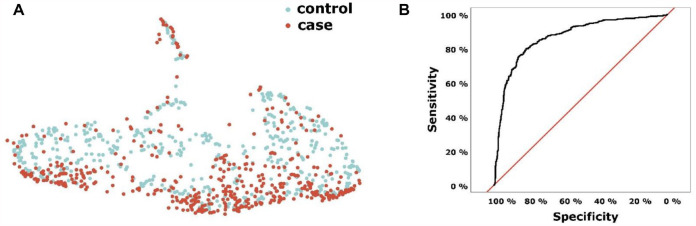
Serum protein profile analyses in PerioGene North cases and controls with (**A**) uniform manifold approximation and projection (UMAP) and (**B**) receiver-operating characteristic (ROC) curve. The ROC curve was based on logit scores from a logistic regression with all proteins as predictors. Logit scores were computed using a leave-one-out cross-validation approach.

The serum protein profile was analyzed using UMAP. A pattern was observed in which cases were overrepresented at the bottom and controls at the top of Figure 1A. ROC analysis showed that the protein profile could separate cases from controls with a sensitivity of 0.81 and specificity of 0.81 (area under the curve [AUC] = 0.87; Fig. 1B).

### Specific Inflammation-Related Proteins Are Associated with Periodontitis and Periodontal Parameters

**Figure 2.**
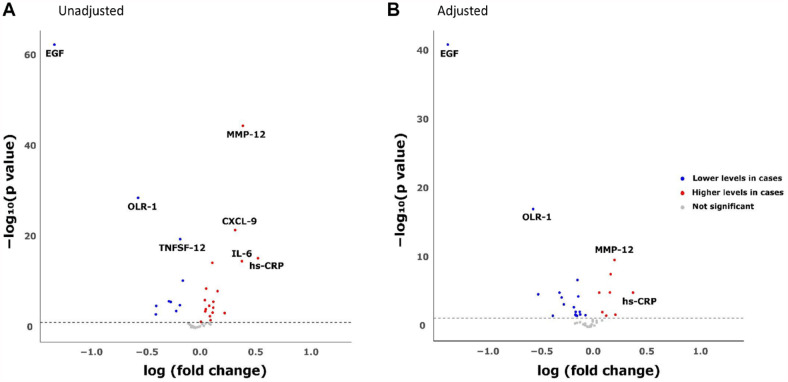
Volcano plot showing significance and fold changes of inflammatory proteins according to (**A**) the nonadjusted regression model and (**B**) the regression model adjusted for age, sex, and ever smoked. The *x*-axis shows the log-fold change, where a positive value indicates higher levels in cases than in controls, whereas a negative value indicates lower levels. The *y*-axis shows the −log^10^ *P* value, where a high value indicate a low *P* value. The dashed line indicates the significance level at 0.05. Significant proteins with higher levels in cases are colored red, and significant proteins with lower levels are colored blue. Not significant proteins are colored gray.

Next, protein levels were analyzed with respect to periodontitis, BoP, PPD, and alveolar bone loss. After adjustment for confounders and multiple testing, the serum levels for hs-CRP and 24 of the 45 analyzed proteins differed significantly between cases and controls (Table 2, left column). In Figure 2A and B, the proteins with the largest effect size and lowest *P* values when comparing cases and controls are highlighted. In the adjusted model (Fig. 2B), EGF and OLR-1 were distinctly separated from the majority with particularly low levels in cases compared with controls and low *P* values. The cases had 3.85 (CI 4.55–3.23, *P* = 7.72e^−44^) times lower serum levels of EGF in comparison with controls and 1.67 times lower levels of OLR-1 (CI 1.89–1.49, *P* = 7.38e^−18^). The levels of hs-CRP showed the highest percentual difference between cases and controls, and MMP-12 displayed both a high difference in concentration and a low *P* value (Fig. 2B). Cases had on average 1.51 times higher levels of hs-CRP (CI 1.28–1.78, *P* = 9.83e^−6^) and 1.27 times higher levels of MMP-12 (CI 1.19–1.36, *P* = 1.84e^−10^) (Table 2, left column).

**Table 2.** Associations of Serum Proteins Significantly Associated with Periodontitis, Gingival Inflammation and Pocket Probing Depth in Cases.

	Case vs. Control (*n* = 478, *n* = 509)		Cases (*n* = 478)			
			Gingival Inflammation^a^ (Low, *n* = 185, vs. High, *n* = 291)		Pocket Probing Depth^b^ (Low, *n* = 75, vs. High, *n* = 203)	
	Exp(B) 95% CI	Adjusted *P* Value	Exp(B) 95% CI	Adjusted *P* Value	Exp(B) 95% CI	Adjusted *P* Value
hs-CRP	1.51 (1.28–1.78)	**<0.001**	1.32 (1.10–1.59)	**0.033**	1.41 (1.08–1.82)	0.151
CCL-2	0.93 (0.88–0.99)	**0.026**	1.02 (0.95–1.09)	0.821	1.01 (0.92–1.11)	0.955
CCL-3	0.76 (0.69–0.85)	**<0.001**	0.94 (0.85–1.06)	0.657	0.99 (0.84–1.16)	0.955
CCL-4	0.89 (0.81–0.97)	**0.024**	1.00 (0.90–1.11)	0.994	1.00 (0.86–1.16)	0.996
CCL-8	0.90 (0.82–0.98)	**0.025**	1.15 (1.04–1.28)	0.056	1.11 (0.96–1.28)	0.670
CCL-13	0.89 (0.82–0.96)	**0.006**	1.05 (0.95–1.15)	0.663	1.04 (0.91–1.19)	0.838
CCL-19	1.13 (1.04–1.23)	**0.009**	1.20 (1.08–1.32)	**0.009**	1.16 (1.01–1.34)	0.466
CSF-1	0.97 (0.95–0.99)	**0.019**	1.01 (0.99–1.04)	0.713	1.00 (0.97–1.04)	0.955
CSF-3	1.23 (1.15–1.31)	**<0.001**	1.18 (1.09–1.28)	**0.001**	1.33 (1.18–1.49)	**<0.001**
CXCL-11	0.88 (0.81–0.97)	**0.017**	1.06 (0.94–1.18)	0.666	1.08 (0.92–1.27)	0.818
CXCL-12	0.91 (0.87–0.95)	**<0.001**	1.06 (1.01–1.12)	0.113	1.05 (0.98–1.13)	0.670
EGF	0.26 (0.22–0.31)	**<0.001**	1.03 (0.81–1.32)	0.958	0.78 (0.55–1.11)	0.670
HGF	0.92 (0.87–0.97)	**0.006**	1.07 (1.00–1.14)	0.273	1.07 (0.97–1.17)	0.670
IL-1β	0.62 (0.51–0.76)	**<0.001**	0.95 (0.75–1.19)	0.821	0.90 (0.65–1.25)	0.838
IL-4	0.79 (0.70–0.89)	**<0.001**	1.15 (1.00–1.31)	0.253	1.08 (0.88–1.31)	0.838
IL-6	1.18 (1.05–1.34)	**0.017**	1.09 (0.95–1.24)	0.567	1.30 (1.08–1.58)	0.111
IL-7	1.22 (1.13–1.32)	**<0.001**	1.15 (1.05–1.27)	**0.033**	1.21 (1.06–1.39)	0.096
IL-13	0.73 (0.56–0.95)	**0.035**	0.81 (0.61–1.09)	0.485	1.04 (0.69–1.59)	0.955
IL-17α	1.28 (1.07–1.54)	**0.018**	1.07 (0.85–1.34)	0.821	1.01 (0.74–1.40)	0.969
MMP-12	1.27 (1.19–1.36)	**<0.001**	1.00 (0.91–1.08)	0.987	1.01 (0.89–1.14)	0.955
OLR-1	0.60 (0.53–0.67)	**<0.001**	1.03 (0.90–1.19)	0.855	0.89 (0.73–1.09)	0.753
OSM	0.78 (0.69–0.87)	**<0.001**	1.05 (0.92–1.21)	0.730	1.11 (0.91–1.35)	0.766
TGFA	0.87 (0.81–0.94)	**0.001**	1.07 (0.97–1.18)	0.473	1.13 (0.98–1.29)	0.670
TNFSF-10	1.10 (1.06–1.15)	**<0.001**	1.07 (1.02–1.12)	0.056	1.14 (1.07–1.22)	**0.007**
TNFSF-12	0.90 (0.87–0.93)	**<0.001**	1.02 (0.97–1.06)	0.713	1.01 (0.95–1.08)	0.929

All associations of proteins are statistically significant between cases and controls after adjustment for multiple testing with the Benjamini and Hochberg method. Exp(B) exponential regression coefficient, 95% CI confidence interval. *P* value based on linear regression model with log protein level as the dependent variable. The model was adjusted for the effects of age, gender, and ever smoked. Six participants, 4 cases, and 2 controls were excluded from the analyses due to missing serum data. CCL, C-C motif chemokine; CI, confidence interval; CSF, granulocyte colony-stimulating factor; CXCL, c-x-c motif chemokine; EGF, epidermal growth factor; HGF, hepatocyte growth factor; hs-CRP, high-sensitivity C-reactive protein; IL, interleukin; MMP, matrix metalloproteinase; OLR-1, oxidized low density lipoprotein receptor 1; OSM, oncostatin M; TGFA, transforming growth factor alpha; TNFSF, tumor necrosis factor ligand superfamily member.

a Low gingival inflammation was defined as bleeding on probing (BoP) <20% and high as BoP ≥20%.

b Low = pocket probing depth (PPD) score of 1.01 to 1.49. High = PPD score of ≥2.00. The score was calculated by assigning each tooth a score of 1 to 3 depending on the degree of PPD. The total score for the entire dentition was summed and then divided by the number of teeth.

Further comparisons showed that hs-CRP (CI 1.10–1.59), C-C motif chemokine 19 (CCL-19; CI 1.08–1.32), granulocyte colony-stimulating factor 3 (CSF-3; CI 1.09–1.28), and interleukin (IL)–7 (CI 1.05–1.27) were significantly higher among cases with high gingival inflammation (BoP ≥20%) (Table 2, middle column).

The levels of CSF-3 (CI 1.18–1.49) and tumor necrosis factor ligand superfamily member (TNFSF)–10 (CI 1.07–1.22) were also higher among cases with high degree of PPD compared with cases with a low degree of PPD (Table 2, right column). No proteins differed significantly among cases with low and high alveolar bone loss. For associations of all analyzed proteins to case and control, gingival inflammation, PPD, and alveolar bone loss, see Appendix Table 4A–D.

### hs-CRP, EGF, OLR-1, and MMP-12 Are Strongly Associated with Severe Periodontitis

**Figure 3.**
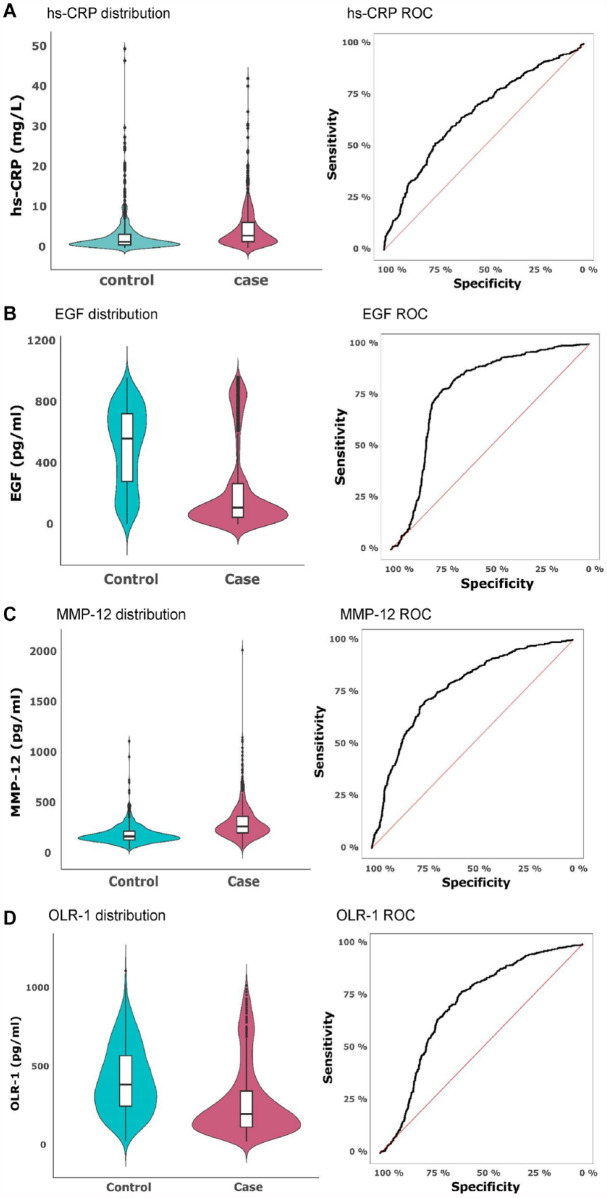
Violin plots demonstrating the distribution of serum levels of (**A**) high-sensitivity C-reactive protein (hs-CRP), (**B**) epidermal growth factor (EGF), (**C**) matrix metalloproteinase–12 (MMP-12), and (**D**) oxidized low-density lipoprotein receptor 1 (OLR-1) in PerioGene North cases and controls. Integrated box plots show median pg/mL values and 25th and 75th percentiles as horizontal lines; whiskers indicate the 10th to 90th percentile. Outliers are plotted as dots. Receiver-operating characteristic (ROC) curve for prediction of cases and controls based on the serum levels of proteins.

The distribution and discriminatory potential of hs-CRP, EGF, OLR-1, and MMP-12 were further evaluated and compared between cases and controls (Fig. 3A–D).

The median level of hs-CRP was 1.7 mg/L (interquartile range [IQR] 0.9–3.5) in controls and 3.2 mg/L in cases (IQR 1.7–6.5). ROC analysis showed that hs-CRP discriminated cases from controls with a sensitivity of 0.74 and a specificity of 0.52 (AUC 0.66) (Fig. 3A). The median level of EGF in cases was 113.8 pg/mL (IQR 49.6–273.2), whereas the median for controls was 565.7 pg/mL (IQR 284.0–728.2). EGF levels could, with a sensitivity of 0.78 and specificity of 0.75, distinguish cases from controls (AUC 0.77) (Fig. 3B). The median level of MMP-12 for cases was 282.6 pg/mL (IQR 216.9–383.6) and for controls was 182.8 pg/mL (IQR 149.3–237.3). MMP-12 could, with a sensitivity of 0.74 and specificity of 0.70, separate cases from controls (AUC 0.77) (Fig. 3C). The median level of OLR-1 was 391.2 pg/mL for controls (IQR 252.3–575.7) and 204.8 pg/mL for cases (IQR 121.5–351.6). ROC analysis showed that levels of OLR-1 could, with a sensitivity of 0.77 and a specificity of 0.60, distinguish cases from controls (AUC 0.71) (Fig. 3D). In a post hoc analysis, the significant findings regarding hs-CRP, EGF, OLR-1, and MMP-12 persisted in all age-stratified 10-y clusters (*P* < 0.05), except for MMP-12 and hs-CRP in the 30- to 40-y age span (see Appendix Table 5).

## Discussion

In this article, we show that periodontitis cases within the PerioGene North study had a distinguishable serum protein profile, with significantly altered levels of 24 individual proteins and hs-CRP. A particularly strong association between high levels of hs-CRP and MMP-12 and low levels of EGF and OLR-1 was observed among the cases. Furthermore, we analyzed and detected associations between specific proteins and periodontal parameters among the cases.

In a systematic review and meta-analysis, Paraskevas and coauthors (2008) provided convincing evidence that individuals with periodontitis have higher levels of hs-CRP (>2.1 mg/L) than controls do (≤2.1 mg/L). In PerioGene North, high hs-CRP levels were associated with periodontitis after adjustment for potential confounders. The median hs-CRP for cases and controls in this study was 3.2 mg/L and 1.7 mg/L, respectively. Our results correspond well to the results presented by Paraskevas et al. (2008). Furthermore, high hs-CRP levels were also associated with having a high degree of gingival inflammation among the cases, indicating a dose-response link between local inflammatory responses and systemic inflammation. This pinpoints periodontitis as a modifiable risk indicator for systemic comorbidity, as previous studies have suggested that hs-CRP levels of greater than 2.03 considerably increase the risk for CVD and can be reversed following treatment (Paraskevas et al. 2008; Li et al. 2017). IL-6, a cytokine known to induce acute phase responses, was also found at higher levels among periodontitis cases in this study. Other cytokines strongly associated with periodontal inflammation and acute phase responses are TNF-α and IL-1β. Regarding TNF-α, ranges between 0.7 pg/mL and 14.6 pg/mL have been reported in diabetic patients with periodontitis in a meta-analysis (Esteves Lima et al. 2021). In the study herein, the cases and control presented with levels of 17.0 and 15.6 pg/mL, respectively, with no significant difference between the groups. Regarding IL-1β, we detected lower levels in cases with periodontitis compared with controls (0.1 in cases and 0.2 pg/mL in controls). Previous studies regarding serum levels of IL-1β and TNF-α in periodontitis presented diverging results and were based on small cohorts with different methods for protein assessment, making comparisons with our results challenging (Gorska et al. 2003; Gumus et al. 2014; Esteves Lima et al. 2021).

MMPs are proteins that play a central role in periodontal inflammation. Interestingly, the cases in this study presented with high levels of MMP-12. As of today, the role of MMP-12 in periodontitis is not understood. However, increasing evidence suggests its involvement in oral diseases (Lin et al. 2023). One study reported that MMP-12 mRNA and protein are elevated in the gingival tissue of periodontitis patients and expressed by cells of monocyte origin (Bjornfot Holmstrom et al. 2017). As MMP-12 exhibits several important functions in tissue remodeling, immune regulation, and wound healing (Bellac et al. 2014; Mouton et al. 2018), its role in periodontitis should be further explored.

Another factor that is crucial for wound healing and tissue homeostasis is EGF. Herein, we demonstrate markedly lower levels of EGF among the cases. This is interesting as EGF per se or as a component in platelet-rich plasma, among other growth factors, can stimulate the proliferation of gingival fibroblasts and periodontal ligament cells in vitro (Nishimura and Terranova 1996; Kim et al. 2011; Poudel et al. 2023). Regarding EGF levels in saliva and gingival crevicular fluid, we found that studies are sparse and provide inconclusive results (Moosavijazi et al. 2014; Wang et al. 2020). One study showing that diabetic patients present with lower salivary EGF levels is intriguing as impaired wound healing is a hallmark of diabetes (Oxford et al. 2000). Therefore, our findings suggest that low EGF levels could represent impaired wound healing and resolution of inflammation, also in periodontitis. Our results are, to the best of our knowledge, the first to show low EGF serum levels in individuals with periodontitis.

Many studies show an association between periodontitis and CVD (Ryden et al. 2016), but the mechanistic link is not entirely understood. We show that periodontitis cases have lower levels of OLR-1, also denoted lectin-like oxidized low-density-lipoprotein receptor (LOX-1). OLR-1 is a receptor for oxidized LDL, expressed on the surface on various cell types or as a soluble protein. Serum OLR-1 levels are positively related to the incidence of CVD, and activation of the receptor is suggested to play a role in atherosclerosis, myocardial fibrosis, and endothelial dysfunction (Barreto et al. 2021). There are studies suggesting that OLR-1 is involved in osteoclastogenesis and peri-implantitis (Ohgi et al. 2018; Zhang et al. 2020), but to our knowledge, serum levels of OLR-1 have not been previously described in relation to periodontitis. Our findings of low OLR-1 levels among cases are intriguing and should be further investigated in other studies.

The major strength of the PerioGene North study is that the study participants are particularly well characterized regarding individual characteristics, periodontal parameters, and general health, as it is based on registrations performed by senior consultants and registry data, respectively. The study was designed in 2003 when the former classification system for periodontitis applied (Armitage 1999). Relative to the classification system used since 2018 (Tonetti et al. 2018a), the cases in PerioGene North are all at stage III or IV, which implies severe periodontitis.

Herein, we confirm the association between periodontitis and previously known risk factors by demonstrating that the cases were more frequently ever-smokers and had a lower education level. Furthermore, the cases more frequently reported heredity for periodontitis, which is line with other reports (Nibali et al. 2019). As the study primarily was designed for genetic studies, the control and case participants were not age matched, which is a limitation of this study. The higher median age among cases might explain why we are unable to show any association between periodontitis and related comorbidities, after adjustment for potential confounders. It is also important to note that the overall prevalence of most general diseases in PerioGene North was low.

We performed an Olink Target 48 Cytokine Panel, a high-throughput multiplex assay by which we obtained absolute concentrations for all analytes, enabling comparison with other studies. However, we were unable to find any other studies that had performed serum inflammatory profiling in large cohorts or any systematic review with meta-analysis on the subject. We would like to acknowledge this gap in knowledge and encourage further studies in this area.

In summary, we present novel information regarding inflammatory-related serum proteins in periodontitis individuals and pinpoint the systemic burden of periodontitis. We present high levels of MMP-12 and low levels of EGF and OLR-1 as interesting candidates that should be further validated in additional cohorts for potential to serve as biomarkers for severe periodontitis. Furthermore, their possible mechanistic role in periodontal inflammation, tissue breakdown, and healing should be experimentally addressed because these could be new targets for treatment.

## Author Contributions

M. Wänman, contributed to conception, design, data acquisition, analysis, and interpretation, drafted and critically revised the manuscript; S. Betnér, A. Esberg, contributed to design, data analysis and interpretation, critically revised the manuscript; C.K. Holm, C. Isehed, A. Holmlund, contributed to data acquisition and interpretation, critically revised the manuscript; P. Palmqvist, contributed to design, data acquisition, critically revised the manuscript; A. Lövgren, contributed to design, data interpretation, critically revised the manuscript; S. Lindquist, contributed to design, data acquisition and interpretation, critically revised the manuscript; L. Hänström, contributed to conception, design, critically revised the manuscript; U.H. Lerner, contributed to conception, design, data interpretation, critically revised the manuscript; E. Kindstedt, contributed to design, data acquisition and interpretation, drafted and critically revised the manuscript; P. Lundberg, contributed to conception, design, data acquisition and interpretation, drafted and critically revised the manuscript. All authors have their final approval and agree to be accountable for all aspects of work.

## Supplemental Material

sj-docx-1-jdr-10.1177_00220345241263320 – Supplemental material for The PerioGene North Study Uncovers Serum Proteins Related to Periodontitis Supplemental material, sj-docx-1-jdr-10.1177_00220345241263320 for The PerioGene North Study Uncovers Serum Proteins Related to Periodontitis by M. Wänman, S. Betnér, A. Esberg, C.K. Holm, C. Isehed, A. Holmlund, P. Palmqvist, A. Lövgren, S. Lindquist, L. Hänström, U.H. Lerner, E. Kindstedt and P. Lundberg in Journal of Dental Research
