# Supplementary material for: The PerioGene North Study Uncovers Serum Proteins Related to Periodontitis
Source: J Dent Res. 2024 Aug 5;103(10):999–1007. doi: 10.1177/00220345241263320 (PMC11402264; doi:10.1177/00220345241263320)
Supplement: sj-docx-1-jdr-10.1177_00220345241263320 – Supplemental material for The PerioGene North Study Uncovers Serum Proteins Related to Periodontitis [file sj-docx-1-jdr-10.1177_00220345241263320.docx]

**Appendix**

**The PerioGene North study uncovers serum proteins related to periodontitis**

**Authors:** Magnus Wänman^1^, Staffan Betnér^2^, Anders Esberg^3^, Cecilia Koskinen Holm^1^, Catrine Isehed^4, 5^, Anders Holmlund^4, 5^, Py Palmqvist^6^, Anna Lövgren^7^, Susanne Lindquist^1,8^, Lennart Hänström^1^, Ulf H. Lerner^1,9^, Elin Kindstedt^1^, Pernilla Lundberg^1^.

**Corresponding author**: Pernilla Lundberg PhD, DDS

**Categorization of Periodontal parameters**

The number of teeth with pocket probing depth (< 4 mm, 4-6 mm and > 6 mm) as well as number of teeth with alveolar bone loss (< 1/3 of the root length, ≥ 1/3 to ≤ 2/3 of the root length and > 2/3 of the root length) were recorded.

According to previous studies, cases were subcategorized by the level of gingival inflammation, periodontal probing depth, and alveolar bone loss (Holmlund et al. 2011).

*Degree of gingival inflammation*: A low degree of gingival inflammation was defined as having BoP < 20%. High degree of gingival inflammation was defined as having BoP ≥ 20%. This was based on previous studies showing that a BoP of 20% or more is associated with an increased risk of progressive tissue destruction (Joss et al. 1994).

*Level of periodontal pocket depth and alveolar bone loss*: Each tooth was given a score of 1-3 depending on the PPD/alveolar bone loss value. A PPD < 4 mm or alveolar bone loss < 1/3 of the root length was given the score 1. A PPD between 4 and 6 mm or alveolar bone loss between ≥ 1/3 to ≤ 2/3 of the root length was given the score 2. A PPD > 6 mm or alveolar bone loss > 2/3 of the root length was given the score 3. The total score for the entire dentition was summed up and then divided by the number of teeth. In the present study, a score between 1.01 - 1.49 was referred to as a low degree of periodontal probing depth or alveolar bone loss, 1.50 – 1.99 as moderate, and over 2.0 as a high level.

**Appendix Table 1.** Proteins analysed in the PerioGene North cohort

|  |  |
| --- | --- |
| ***Protein full name*** | ***Abbreviation*** |
|  |  |
| High sensitivity C reactive protein | hs-CRP |
| C-C motif chemokine 2 | CCL-2 |
| C-C motif chemokine 3 | CCL-3 |
| C-C motif chemokine 4 | CCL-4 |
| C-C motif chemokine 7 | CCL-7 |
| C-C motif chemokine 8 | CCL-8 |
| Eotaxin | CCL-11 |
| C-C motif chemokine 13 | CCL-13 |
| C-C motif chemokine 19 | CCL-19 |
| Macrophage colony-stimulating factor | CSF-1 |
| Granulocyte-macrophage colony stimulating factor | CSF-2 |
| Granulocyte colony-stimulating factor | CSF-3 |
| C-X-C motif chemokine 8 | CXCL-8 |
| C-X-C motif chemokine 9 | CXCL-9 |
| C-X-C motif chemokine 10 | CXCL-10 |
| C-X-C motif chemokine 11 | CXCL-11 |
| Stromal cell-derived factor 1 | CXCL-12 |
| Pro-epidermal growth factor | EGF |
| Fms-raletad tyrosine kinase 3 ligand | FLT3LG |
| Hepatocyte growth factor | HGF |
| Interferon gamma | IFNG |
| Interleukin-1 beta | IL-1B |
| Interleukin-2 | IL-2 |
| Interleukin-4 | IL-4 |
| Interleukin-6 | IL-6 |
| Interleukin-7 | IL-7 |
| Interleukin-10 | IL-10 |
| Interleukin-13 | IL-13 |
| Interleukin-15 | IL-15 |
| Interleukin-17α | IL-17A |
| Interleukin-17C | IL-17C |
| Interleukin-17F | IL-17F |
| Interleukin-18 | IL-18 |
| Interleukin-27 | IL-27 |
| Interleukin-33 | IL-33 |
| Lymphotoxin-alpha | LTA |
| Interstitial collagenase | MMP-1 |
| Macrophage metalloelastase | MMP-12 |
| Oxidized low-density lipoprotein receptor 1 | OLR-1 |
| Oncostatin-M | OSM |
| Protransforming growth factor alpha | TGFA |
| Tumor necrosis factor | TNF-α |
| Tumor necrosis factor ligand superfamily member 10 | TNFSF-10 |
| Tumor necrosis factor ligand superfamily member 12 | TNFSF-12 |
| Thymic stromal lymphopoietin | TSLP |
| Vascular endothelial growth factor A | VEGFA |
|  |  |

**Appendix Table 2.** Number of missing observations for analysed variables in PerioGene North

|  |  |
| --- | --- |
| **Variable** | **Number of missing observations** |
|  |  |
| Case or control | 0 |
| Age at examination | 0 |
| Sex | 0 |
| BMI | 50 |
| Education level | 1 |
| Tobacco usage | 0 |
| Self-reported heredity of periodontitis | 0 |
| Country of birth | 0 |
| Number of teeth | 0 |
| Bleeding on probing % | 2 |
| n teeth with pocket probing depth | 0 |
| n teeth with alveolar bone loss | 0 |
| BoP level group | 2 |
| PPD level group | 0 |
| Alveolar bone loss level group | 0 |
| General diseases | 0 |
| Protein concentration | 6 |
|  |  |

**Appendix Table 3.** Associations of general diseases to case and control.

|  |  |  |
| --- | --- | --- |
| **General diseases (ICD-10 code)** | **OR (95%CI)** | ***P*-value^a^** |
|  |  |  |
| Cancer *(C0-99)* | 0.8 (0.4-2.0) | 0.748 |
| Diabetes type 1 *(E10)* | 2.9 (0.6-14.3) | 0.187 |
| Diabetes type 2 *(E11)* | 3.7 (0.7-18.9) | 0.123 |
| Obesity *(E65-66)* | 2.6 (0.8-8.3) | 0.121 |
| Cardiovascular disease^b^ (*I0-99)* | 1.0 (0.6-1.6) | 0.970 |
| High blood pressure *(I10-15)* | 1.3 (0.7-2.5) | 0.397 |
| Ischemic heart disease *(I20-25)* | 2.8 (0.8-10.3) | 0.114 |
| Cerebrovascular conditions *(I60-69)* | 2.7 (0.4-18.1) | 0.301 |
| Lung disease *(J40-45)* | 0.8 (0.3-1.8) | 0.534 |
| Rheumatoid arthritis *(M05-06)* | 1.7 (0.1-23.0) | 0.604 |
| SLE *(M32)* | NA | NA |
| Osteoporosis *(M80-81)* | 2.2 (0.2-26.5) | 0.544 |
| Inflammatory bowel disease *(K50-55)* | 1.1 (0.3-4.3) | 0.886 |
|  |  |  |

1. *P*-value based on logistic regression with case/control as dependent variable. Adjusted for the effects of age, gender and ever smoked.
2. Cardiovascular disease is a merged category for high blood pressure, ischemic heart disease and cerebrovascular conditions.

**Appendix Table 4A -D.** Comparison of protein concentrations in A. case and control, B. gingival inflammation level, C. PPD level and D. alveolar bone loss level.

1. Comparison of protein concentrations in cases and controls

|  | **Control** | **Case** | **Case versus control^a^** | |
| --- | --- | --- | --- | --- |
|  | *N=509* | *N=478* |  |  |
|  |  |  |  | |
| *Protein (pg/mL)* | *Median controls*  *(IQR)* | *Median cases*  *(IQR)* | *Exp(B)*  *95 % CI* | *Adjusted*  *P-value* |
|  |  |  |  |  |
| hs-CRP^b^ | 1.7 (0.9-3.5) | 3.2 (1.7-6.5) | 1.51 (1.28–1.78) | **9.83e-6** |
| CCL-2 | 520.7 (420.5-644.5) | 530.2 (421.7-664.6) | 0.93 (0.88-0.99) | **2.64e-2** |
| CCL-3 | 8.4 (5.8-14.7) | 7.4 (5.7-10.8) | 0.76 (0.69-0.85) | **9.83e-6** |
| CCL-4 | 118.5 (89.2-169.2) | 115.3 (85.3-158.6) | 0.89 (0.81-0.97) | **2.35e-2** |
| CCL-7 | 0.9 (0.7-1.3) | 1.0 (0.8-1.5) | 0.90 (0.76-1.05) | 2.55e-1 |
| CCL-8 | 60.6 (44.2-78.7) | 56.9 (39.7-78.6) | 0.90 (0.82-0.98) | **2.45e-2** |
| CCL-11 | 125.8 (100.8-155.6) | 134.6 (108.8-168.5) | 0.97 (0.92-1.02) | 3.27e-1 |
| CCL-13 | 182.0 (135.5-237.5) | 173.4 (123.2-236.5) | 0.89 (0.82-0.96) | **6.32e-3** |
| CCL-19 | 87.6 (70.0-113.2) | 104.1 (79.2-145.5) | 1.13 (1.04-1.23) | **8.67e-3** |
| CSF-1 | 128.2 (116.5-140.6) | 128.7 (117.9-140.6) | 0.97 (0.95-0.99) | **1.97e-2** |
| CSF-2 | 0.2 (0.1-0.2) | 0.2 (0.1-0.2) | 0.95 (0.86-1.05) | 4.10e-1 |
| CSF-3 | 95.2 (76.6-122.2) | 108.3 (87.1-145.7) | 1.23 (1.15-1.31) | **2.19e-8** |
| CXCL-8 | 13.4 (10.0-24.8) | 15.3 (11.2-21.6) | 1.08 (0.89-1.31) | 5.10e-1 |
| CXCL-9 | 46.5 (35.2-62.2) | 66.1 (47.2-98.6) | 1.07 (0.99-1.16) | 1.60e-1 |
| CXCL-10 | 95.7 (73.6-134.0) | 116.5 (84.5-166.5) | 0.96 (0.88-1.05) | 4.62e-1 |
| CXCL-11 | 61.4 (47.1-91.5) | 61.1 (41.3-84.2) | 0.88 (0.81-0.97) | **1.70e-2** |
| CXCL-12 | 214.3 (182.1-249.0) | 187.8 (158.6-229.0) | 0.91 (0.87-0.95) | **3.61e-5** |
| EGF | 565.7 (284.0-728.2) | 113.8 (49.6-273.2) | 0.26 (0.22-0.31) | **7.72e-44** |
| FLT3LG | 110.4 (91.7-132.2) | 128.6 (103.9-156.1) | 1.04 (0.99-1.09) | 1.60e-1 |
| HGF | 551.1 (437.2-666.3) | 532.4 (421.7-649.5) | 0.92 (0.87-0.97) | **6.32e-3** |
| IFNG | 0.2 (0.1-0.3) | 0.2 (0.2-0.4) | 1.02 (0.92-1.13) | 7.73e-1 |
| IL-1β | 0.2 (0.1-0.3) | 0.1 (0.1-0.2) | 0.62 (0.51-0.76) | **1.60e-5** |
| IL-2 | 0.0 (0.0-0.0) | 0.0 (0.0-0.0) | 1.01 (0.89-1.14) | 8.98e-1 |
| IL-4 | 0.0 (0.0-0.0) | 0.0 (0.0-0.0) | 0.79 (0.70-0.89) | **4.91e-4** |
| IL-6 | 1.8 (1.3-2.8) | 3.0 (2.0-4.6) | 1.18 (1.05-1.34) | **1.74e-2** |
| IL-7 | 4.9 (3.7-6.7) | 6.2 (4.2-8.4) | 1.22 (1.13-1.32) | **9.73e-6** |
| IL-10 | 6.4 (4.8-8.6) | 7.3 (5.1-10.1) | 1.08 (0.97-1.19) | 2.33e-1 |
| IL-13 | 0.2 (0.1-0.7) | 0.2 (0.1-0.5) | 0.73 (0.56-0.95) | **3.47e-2** |
| IL-15 | 12.2 (10.8-13.9) | 13.6 (11.6-15.7) | 1.04 (1.00-1.08) | 9.48e-2 |
| IL-17α | 0.3 (0.2-0.6) | 0.4 (0.2-0.8) | 1.28 (1.07-1.54) | **1.84e-2** |
| IL-17C | 13.3 (9.6-18.0) | 13.3 (9.1-19.8) | 0.96 (0.88-1.06) | 5.03e-1 |
| IL-17F | 0.7 (0.4-1.2) | 0.6 (0.4-1.2) | 1.02 (0.86-1.20) | 8.75e-1 |
| IL-18 | 274.0 (221.4-330.3) | 294.0 (237.3-370.7) | 1.03 (0.97-1.08) | 4.10e-1 |
| IL-27 | 4.9 (3.1-7.3) | 5.9 (3.4-8.8) | 1.13 (0.99-1.29) | 1.12e-1 |
| IL-33 | 0.1 (0.1-0.1) | 0.1 (0.1-0.2) | 1.03 (0.88-1.21) | 7.73e-1 |
| LTA | 7.6 (6.4-9.1) | 7.7 (6.4-9.2) | 1.01 (0.97-1.06) | 6.86e-1 |
| MMP-1 | 2908.4 (1765.5-4340.0) | 2802.7 (1744.0-4297.4) | 0.92 (0.83-1.03) | 2.03e-1 |
| MMP-12 | 182.8 (149.3-237.3) | 282.6 (216.9-383.6) | 1.27 (1.19-1.36) | **1.84e-10** |
| OLR-1 | 391.2 (252.3-575.7) | 204.8 (121.5-351.6) | 0.60 (0.53-0.67) | **7.38e-18** |
|  |  |  |  |  |
|  | **Control** | **Case** | **Case versus control^a^** | |
|  | *N=509* | *N=478* |  |  |
|  |  |  |  | |
| *Protein*  *pg/mL)* | *Median controls*  *(IQR)* | *Median cases*  *(IQR)* | *Exp(B)*  *95 % CI* | *Adjusted*  *P-value* |
|  |  |  |  |  |
| OSM | 8.7 (5.9-12.1) | 6.5 (4.4-9.9) | 0.78 (0.69-0.87) | **5.20e-5** |
| TGFA | 17.0 (12.5-23.4) | 14.9 (10.3-21.6) | 0.87 (0.81-0.94) | **1.35e-3** |
| TNF-α | 15.6 (13.6-18.9) | 17.0 (14.0-19.9) | 1.00 (0.95-1.06) | 8.98e-1 |
| TNFSF-10 | 450.4 (388.8-523.2) | 483.4 (415.7-571.3) | 1.10 (1.06-1.15 | **9.83e-6** |
| TNFSF-12 | 784.6 (676.2-903.2) | 676.8 (583.8-807.6) | 0.90 (0.87-0.93) | **1.50e-7** |
| TSLP | 0.1 (0.0-0.1) | 0.1 (0.0-0.1) | 0.91 (0.73-1.13) | 4.79e-1 |
| VEGFA | 534.8 (378.8-785.9) | 554.5 (388.8-836.3) | 0.99 (0.91-1.08) | 8.25e-1 |
|  |  |  |  |  |

Abbreviations: *Exp(B)* exponential regression coefficient, *95% CI* confidence interval

*P*-value based on linear regression model with log protein level as the dependent variable. The model was adjusted for the effects of age, gender and ever smoked

a) 6 participants, 4 cases and 2 controls excluded from analyses due to missing serum data.

b) hs-CRP measured in mg/L

1. Comparison of protein concentrations in low and high levels of gingival inflammation among cases

|  | **Cases (N=478)^a^** | | | **Low versus high**  **gingival inflammation** | |
| --- | --- | --- | --- | --- | --- |
|  | *N=185* | *N=291* | |  |  |
|  |  | |  |  | |
| *Protein (pg/mL)* | *Low gingival inflammation*  *median (IQR)* | | *High gingival inflammation median (IQR)* | *Exp(B)*  *95 % CI* | *Adjusted*  *P-value* |
|  |  | |  |  |  |
| hs-CRP^b^ | 2.8 (1.5-5.1) | | 3.4 (1.7-7.3) | 1.32 (1.10-1.59) | **0.033** |
| CCL-2 | 531.8 (412.9-681.3) | | 524.0 (418.9-638.3) | 1.02 (0.95-1.09) | 0.821 |
| CCL-3 | 7.4 (5.7-11.3) | | 7.4 (5.8-10.6) | 0.94 (0.85-1.06) | 0.657 |
| CCL-4 | 116.6 (83.4-156.5) | | 114.7 (86.6-160.0) | 1.00 (0.90-1.11) | 0.994 |
| CCL-7 | 0.9 (0.7-1.4) | | 1.1 (0.8-1.5) | 0.95 (0.80-1.14) | 0.821 |
| CCL-8 | 52.8 (37.5-71.4) | | 58.0 (41.0-83.2) | 1.15 (1.04-1.28) | 0.056 |
| CCL-11 | 132.8 (110.2-167.8) | | 134.7 (108.6-168.9) | 1.00 (0.94.1.07) | 0.994 |
| CCL-13 | 170.7 (118.4-246.0) | | 173.5 (126.2-226.9) | 1.05 (0.95-1.15) | 0.663 |
| CCL-19 | 94.6 (73.7-130.7) | | 109.6 (82.4-158.3) | 1.20 (1.08-1.32) | **0.009** |
| CSF-1 | 129.5 (117.2-141.4) | | 128.4 (117.9-139.6) | 1.01 (0.99-1.04) | 0.713 |
| CSF-2 | 0.2 (0.1-0.2) | | 0.2 (0.1-0.2) | 0.99 (0.88-1.11) | 0.969 |
| CSF-3 | 101.1 (81.1-127.4) | | 111.9 (88.5-157.9) | 1.18 (1.09-1.28) | **0.001** |
| CXCL-8 | 16.8 (11.5-25.8) | | 14.5 (11.0-20.7) | 0.90 (0.73-1.13) | 0.666 |
| CXCL-9 | 70.3 (51.0-99.4) | | 64.1 (44.8-98.5) | 0.99 (0.90-1.09) | 0.987 |
| CXCL-10 | 117.0 (84.5-164.2) | | 114.7 (84.8-167.5) | 1.10 (0.99-1.23) | 0.288 |
| CXCL-11 | 60.0 (39.7-82.0) | | 61.1 (42.5-86.2) | 1.06 (0.94-1.18) | 0.666 |
| CXCL-12 | 181.5 (149.3-228.6) | | 191.9 (164.9-229.8) | 1.06 (1.01-1.12) | 0.113 |
| EGF | 107.3 (42.4-437.6) | | 116.6 (55.0-245.7) | 1.03 (0.81-1.32) | 0.958 |
| FLT3LG | 133.5 (106.2-165.0) | | 126.3 (101.5-152.0) | 0.96 (0.91-1.01) | 0.455 |
| HGF | 497.2 (407.1-632.1) | | 540.9 (438.3-653.8) | 1.07 (1.00-1.14) | 0.273 |
| IFNG | 0.2 (0.2-0.4) | | 0.2 (0.2-0.3) | 1.01 (0.89-1.15) | 0.987 |
| IL-1β | 0.1 (0.1-0.3) | | 0.1 (0.1-0.2) | 0.95 (0.75-1.19) | 0.821 |
| IL-2 | 0.0 (0.0-0.0) | | 0.0 (0.0-0.0) | 1.00 (0.87-1.14) | 0.994 |
| IL-4 | 0.0 (0.0-0.0) | | 0.0 (0.0-0.0) | 1.15 (1.00-1.31) | 0.253 |
| IL-6 | 2.8 (1.8-4.7) | | 3.0 (2.1-4.6) | 1.09 (0.95-1.24) | 0.567 |
| IL-7 | 6.1 (3.4-8.0) | | 6.4 (4.7-9.0) | 1.15 (1.05-1.27) | **0.033** |
| IL-10 | 7.2 (4.9-10.1) | | 7.3 (5.2-10.1) | 1.05 (0.94-1.19) | 0.666 |
| IL-13 | 0.2 (0.1-0.6) | | 0.2 (0.1-0.4) | 0.81 (0.61-1.09) | 0.485 |
| IL-15 | 13.2 (11.2-15.5) | | 13.7 (11.7-15.7) | 1.04 (0.99-1.09) | 0.386 |
| IL-17α | 0.4 (0.2-0.8) | | 0.4 (0.2-0.8) | 1.07 (0.85-1.34) | 0.821 |
| IL-17C | 12.5 (8.2-18.7) | | 13.7 (9.7-20.8) | 1.18 (1.05-1.32) | 0.056 |
| IL-17F | 0.8 (0.4-1.4) | | 0.6 (0.4-1.0) | 0.88 (0.73-1.08) | 0.567 |
| IL-18 | 293.7 (237.7-365.1) | | 293.4 (236.7-370.9) | 0.99 (0.93-1.05) | 0.865 |
| IL-27 | 5.8 (3.3-8.8) | | 5.9 (3.4-9.0) | 0.98 (0.83-1.14) | 0.912 |
| IL-33 | 0.1 (0.1-0.2) | | 0.1 (0.1-0.2) | 1.11 (0.92-1.33) | 0.582 |
| LTA | 7.8 (6.5-9.2) | | 7.6 (6.4-9.1) | 0.99 (0.94-1.04) | 0.821 |
| MMP-1 | 2751.0 (1509.7-4297.0) | | 2840.8 (1812.0-4372.2) | 1.08 (0.95-1.23) | 0.567 |
| MMP-12 | 289.4 (224.2-378.8) | | 275.0 (209.0-387.6) | 1.00 (0.91-1.08) | 0.987 |
| OLR-1 | 187.2 (115.6-437.9) | | 211.4 (132.5-329.5) | 1.03 (0.90-1.19) | 0.855 |
|  |  | |  |  |  |
|  | **Cases (N=478)^a^** | | | **Low versus high**  **gingival inflammation** | |
|  | *N=185* | *N=291* | |  |  |
|  |  | |  |  | |
| *Protein*  *pg/mL)* | *Low gingival inflammation*  *median (IQR)* | | *High gingival inflammation*  *median (IQR)* | *Exp(B)*  *95 % CI* | *Adjusted*  *P-value* |
|  |  | |  |  |  |
| OSM | 6.1 (3.7-10.3) | | 6.8 (4.8-9.6) | 1.05 (0.92-1.21) | 0.730 |
| TGFA | 13.8 (9.7-20.2) | | 15.2 (10.9-22.5) | 1.07 (0.97-1.18) | 0.473 |
| TNF-α | 17.0 (13.6-20.2) | | 16.9 (14.2-19.5) | 1.02 (0.96-1.07) | 0.800 |
| TNFSF-10 | 458.5 (401.5-540.9) | | 502.9 (435.1-588.5) | 1.07 (1.02-1.12) | 0.056 |
| TNFSF-12 | 660.7 (579.5-787.5) | | 687.2 (586.3-815.6) | 1.02 (0.97-1.06) | 0.713 |
| TSLP | 0.1 (0.0-0.1) | | 0.1 (0.0-0.1) | 0.82 (0.64-1.06) | 0.435 |
| VEGFA | 544.0 (389.7-801.0) | | 570.8 (389.7-863.8) | 1.06 (0.96-1.18) | 0.567 |
|  |  | |  |  |  |

Abbreviations: *Exp(B)* exponential regression coefficient, *95% CI* confidence interval

*P*-value based on linear regression model with log protein level as the dependent variable. The model was adjusted for the effects of age, gender and ever smoked.

a) 4 participants excluded from analyses due to missing serum data.

b) hs-CRP measured in mg/L

1. Comparison of protein concentrations in low and high levels of PPD among the cases

|  | **Cases (N=478)^a^** | | **Low versus high**  **PPD** | |
| --- | --- | --- | --- | --- |
|  | *N=75* | *N=203* |  |  |
|  |  |  |  | |
| *Protein (pg/mL)* | *Low PPD*  *median (IQR)* | *High PPD*  *median (IQR)* | *Exp(B)*  *95 % CI* | *Adjusted*  *P-value* |
|  |  |  |  |  |
| hs-CRP^b^ | 2.8 (1.5-5.8) | 3.4 (1.7-7.4) | 1.41 (1.08-1.82) | 0.151 |
| CCL-2 | 530.2 (460.0-632.1) | 521.5 (406.1-649.1) | 1.01 (0.92-1.11) | 0.955 |
| CCL-3 | 7.7 (5.9-11.9) | 7.2 (5.7-10.1) | 0.99 (0.84-1.16) | 0.955 |
| CCL-4 | 116.3 (85.9-152.9) | 113.5 (82.6-155.3) | 1.00 (0.86-1.16) | 0.996 |
| CCL-7 | 0.9 (0.7-1.5) | 1.0 (0.8-1.5) | 1.01 (0.78-1.31) | 0.969 |
| CCL-8 | 57.2 (41.4-71.4) | 61.6 (43.2-83.0) | 1.11 (0.96-1.28) | 0.670 |
| CCL-11 | 139.2 (115.2-176.8) | 133.1 (104.7-171.2) | 0.97 (0.88-1.06) | 0.838 |
| CCL-13 | 173.5 (127.1-216.5) | 164.8 (116.4-235.4) | 1.04 (0.91-1.19) | 0.838 |
| CCL-19 | 98.6 (75.2-130.4) | 110.3 (84.5-151.4) | 1.16 (1.01-1.34) | 0.466 |
| CSF-1 | 131.1 (117.0-142.8) | 127.5 (117.4-138.8) | 1.00 (0.97-1.04) | 0.955 |
| CSF-2 | 0.2 (0.1-0.3) | 0.2 (0.1-0.2) | 0.92 (0.78-1.08) | 0.766 |
| CSF-3 | 89.0 (71.4-117.3) | 117.5 (93.4-159.9) | 1.33 (1.18-1.49) | **< 0.001** |
| CXCL-8 | 17.6 (11.5-43.1) | 14.6 (11.2-20.1) | 0.61 (0.45-0.84) | 0.063 |
| CXCL-9 | 69.5 (47.8-98.5) | 64.5 (43.6-94.7) | 1.02 (0.89-1.17) | 0.929 |
| CXCL-10 | 116.7 (87.2-178.1) | 115.7 (79.0-168.0) | 1.11 (0.95-1.29) | 0.670 |
| CXCL-11 | 61.8 (41.5-75.8) | 62.4 (41.0-84.1) | 1.08 (0.92-1.27) | 0.818 |
| CXCL-12 | 179.2 (149.3-220.5) | 190.6 (162.4-229.9) | 1.05 (0.98-1.13) | 0.670 |
| EGF | 124.6 (48.5-660.3) | 111.4 (57.3-234.1) | 0.78 (0.55-1.11) | 0.670 |
| FLT3LG | 126.3 (104.2-153.0) | 127.5 (101.2-154.8) | 1.01 (0.94-1.09) | 0.929 |
| HGF | 508.2 (383.0-649.4) | 534.8 (425.6-641.7) | 1.07 (0.97-1.17) | 0.670 |
| IFNG | 0.2 (0.2-0.4) | 0.2 (0.2-0.3) | 0.97 (0.81-1.16) | 0.929 |
| IL-1β | 0.1 (0.1-0.4) | 0.1 (0.1-0.2) | 0.90 (0.65-1.25) | 0.838 |
| IL-2 | 0.0 (0.0-0.0) | 0.0 (0.0-0.0) | 1.07 (0.89-1.29) | 0.838 |
| IL-4 | 0.0 (0.0-0.0) | 0.0 (0.0-0.0) | 1.08 (0.88-1.31) | 0.838 |
| IL-6 | 2.5 (1.6-4.5) | 3.0 (2.1-5.0) | 1.30 (1.08-1.58) | 0.111 |
| IL-7 | 6.0 (3.0-7.9) | 6.6 (4.6-9.2) | 1.21 (1.06-1.39) | 0.096 |
| IL-10 | 6.8 (5.0-9.5) | 7.5 (5.1-10.1) | 1.11 (0.94-1.32) | 0.670 |
| IL-13 | 0.2 (0.1-0.5) | 0.2 (0.1-0.5) | 1.04 (0.69-1.59) | 0.955 |
| IL-15 | 13.1 (10.9-15.3) | 14.0 (12.0-15.7) | 1.05 (0.98-1.12) | 0.670 |
| IL-17α | 0.4 (0.2-1.0) | 0.4 (0.2-0.9) | 1.01 (0.74-1.40) | 0.969 |
| IL-17C | 13.1 (8.9-19.9) | 13.9 (9.4-22.3) | 1.11 (0.94-1.31) | 0.670 |
| IL-17F | 0.7 (0.3-1.2) | 0.7 (0.4-1.3) | 1.10 (0.83-1.46) | 0.838 |
| IL-18 | 265.8 (223.6-339.6) | 301.6 (239.9-387.9) | 1.08 (0.99-1.18) | 0.670 |
| IL-27 | 5.6 (3.2-8.6) | 5.9 (3.4-8.7) | 1.08 (0.86-1.35) | 0.838 |
| IL-33 | 0.1 (0.0-0.2) | 0.1 (0.1-0.2) | 1.28 (0.99-1.66) | 0.585 |
| LTA | 8.0 (6.7-9.3) | 7.5 (6.2-9.0) | 0.95 (0.88-1.03) | 0.670 |
| MMP-1 | 2904.8 (1832.4-4297.3) | 2812.2 (1816.6-4297.3) | 1.02 (0.85-1.22) | 0.955 |
| MMP-12 | 273.9 (200.6-361.6) | 271.3 (204.0-386.4) | 1.01 (0.89-1.14) | 0.955 |
| OLR-1 | 194.9 (119.1-489.9) | 202.5 (121.4-319.0) | 0.89 (0.73-1.09) | 0.753 |
|  |  |  |  |  |
|  | **Cases (N=478)^a^** | | **Low versus high**  **PPD** | |
|  | *N=75* | *N=203* |  |  |
|  |  |  |  | |
| *Protein*  *pg/mL)* | *Low PPD*  *Median (IQR)* | *High PPD*  *Median (IQR)* | *Exp(B)*  *95 % CI* | *Adjusted*  *P-value* |
|  |  |  |  |  |
| OSM | 5.5 (3.6-10.7) | 6.5 (4.8-9.4) | 1.11 (0.91-1.35) | 0.766 |
| TGFA | 12.7 (10.6-18.0) | 15.5 (10.4-22.8) | 1.13 (0.98-1.29) | 0.670 |
| TNF-α | 16.6 (14.5-20.3) | 17.2 (13.9-19.8) | 1.06 (0.98-1.15) | 0.670 |
| TNFSF-10 | 437.4 (383.6-500.1) | 497.1 (432.8-589.1) | 1.14 (1.07-1.22) | **0.007** |
| TNFSF-12 | 679.5 (592.1-798.1) | 705.1 (595.8-825.1) | 1.01 (0.95-1.08) | 0.929 |
| TSLP | 0.1 (0.0-0.2) | 0.1 (0.0-0.1) | 0.71 (0.49-1.03) | 0.644 |
| VEGFA | 587.6 (457.0-789.1) | 599.4 (368.70-920.6) | 1.07 (0.92-1.24) | 0.838 |
|  |  |  |  |  |

Abbreviations: *Exp(B)* exponential regression coefficient, *95% CI* confidence interval

*P*-value based on linear regression model with log protein level as the dependent variable. The model was adjusted for the effects of age, gender and ever smoked.

a) 4 participants excluded from analyses due to missing serum data.

b) hs-CRP measured in mg/L

1. Comparison of protein concentrations in low and high levels of alveolar bone loss among the cases

|  | **Cases (N=478)^a^** | | **Low versus high**  **alveolar bone loss** | |
| --- | --- | --- | --- | --- |
|  | *N=117* | *N=115* |  |  |
|  |  |  |  | |
| *Protein (pg/mL)* | *Low alveolar bone loss*  *median (IQR)* | *High alveolar bone loss*  *median (IQR)* | *Exp(B)*  *95 % CI* | *Adjusted*  *P-value* |
|  |  |  |  |  |
| hs-CRP^b^ | 3.2 (1.3-6.3) | 3.3 (1.6-7.3) | 1.06 (0.82-1.37) | 0.854 |
| CCL-2 | 502.4 (403.2-622.4) | 536.7 (431.9-680.6) | 1.02 (0.93-1.12) | 0.875 |
| CCL-3 | 7.2 (6.0-10.6) | 7.9 (5.9-11.3) | 1.10 (0.94-1.29) | 0.775 |
| CCL-4 | 116.6 (85.2-155.0) | 117.9 (86.2-171.9) | 1.09 (0.94-1.26) | 0.775 |
| CCL-7 | 1.0 (0.7-1.5) | 1.0 (0.8-1.5) | 1.13 (0.88-1.47) | 0.783 |
| CCL-8 | 53.1 (41.0-75.3) | 59.8 (39.8-83.7) | 1.04 (0.90-1.20) | 0.854 |
| CCL-11 | 130.1 (104.0-155.9) | 139.8 (110.9-178.7) | 1.06 (0.97-1.16) | 0.775 |
| CCL-13 | 165.6 (122.0-237.2) | 173.1 (115.8-229.7) | 1.01 (0.88-1.15) | 0.989 |
| CCL-19 | 98.7 (79.5-142.4) | 110.3 (84.1-150.0) | 1.05 (0.92-1.21) | 0.839 |
| CSF-1 | 129.3 (113.0-140.6) | 128.7 (121.1-140.6) | 1.03 (0.99-1.06) | 0.775 |
| CSF-2 | 0.2 (0.1-0.2) | 0.2 (0.1-0.2) | 1.01 (0.86-1.19) | 0.989 |
| CSF-3 | 104.4 (84.4-138.5) | 112.0 (88.4-156.4) | 1.14 (1.01-1.27) | 0.613 |
| CXCL-8 | 15.3 (11.3-24.7) | 15.9 (11.3-24.0) | 1.07 (0.78-1.46) | 0.854 |
| CXCL-9 | 64.1 (45.6-84.6) | 62.1 (46.6-95.5) | 1.08 (0.95-1.24) | 0.775 |
| CXCL-10 | 115.1 (84.6-152.4) | 113.6 (78.3-167.0) | 1.06 (0.91-1.24) | 0.820 |
| CXCL-11 | 57.3 (40.2-83.1) | 62.3 (46.3-84.5) | 1.14 (0.97-1.34) | 0.739 |
| CXCL-12 | 181.5 (149.3-225.0) | 190.6 (162.1-229.9) | 1.05 (0.98-1.13) | 0.775 |
| EGF | 124.6 (59.5-305.7) | 105.5 (43.7-433.9) | 0.82 (0.59-1.16) | 0.775 |
| FLT3LG | 120.7 (104.0-150.1) | 128.5 (101.4-156.5) | 1.00 (0.92-1.07) | 0.989 |
| HGF | 522.8 (447.2-631.2) | 550.9 (439.4-654.8) | 1.03 (0.94-1.13) | 0.845 |
| IFNG | 0.2 (0.2-0.4) | 0.2 (0.2-0.3) | 1.00 (0.83-1.20) | 0.993 |
| IL-1B | 0.1 (0.1-0.2) | 0.2 (0.1-0.3) | 1.36 (0.98-1.87) | 0.730 |
| IL-2 | 0.0 (0.0-0.0) | 0.0 (0.0-0.0) | 1.10 (0.92-1.33) | 0.775 |
| IL-4 | 0.0 (0.0-0.0) | 0.0 (0.0-0.0) | 1.04 (0.86-1.26) | 0.854 |
| IL-6 | 2.5 (1.8-3.9) | 3.4 (2.0-4.7) | 1.18 (0.98-1.42) | 0.739 |
| IL-7 | 6.0 (4.1-8.3) | 6.2 (3.9-8.7) | 1.04 (0.91-1.19) | 0.845 |
| IL-10 | 6.9 (4.8-10.1) | 7.0 (5.1-9.4) | 0.99 (0.84-1.17) | 0.989 |
| IL-13 | 0.1 (0.1-0.5) | 0.2 (0.1-0.4) | 0.95 (0.63-1.44) | 0.934 |
| IL-15 | 13.2 (11.2-15.1) | 13.9 (11.7-15.8) | 1.05 (0.98-1.12) | 0.775 |
| IL-17A | 0.4 (0.2-0.6) | 0.5 (0.2-1.0) | 1.37 (1.00-1.87) | 0.730 |
| IL-17C | 12.2 (8.9-16.7) | 14.2 (10.6-23.5) | 1.28 (1.08-1.50) | 0.339 |
| IL-17F | 0.7 (0.4-1.4) | 0.7 (0.4-1.3) | 0.93 (0.70-1.22) | 0.845 |
| IL-18 | 280.3 (223.6-359.5) | 304.7 (237.3-373.8) | 1.04 (0.95-1.13) | 0.839 |
| IL-27 | 5.7 (3.2-8.9) | 6.3 (3.9-9.2) | 1.14 (0.91-1.42) | 0.775 |
| IL-33 | 0.1 (0.1-0.2) | 0.1 (0.1-0.2) | 1.06 (0.82-1.37) | 0.854 |
| LTA | 7.7 (6.5-9.2) | 7.2 (6.1-8.7) | 1.00 (0.93-1.08) | 0.989 |
| MMP-1 | 2807.4 (1624.7-4593.4) | 2636.2 (1740.1-3826.2) | 0.94 (0.79-1.13) | 0.839 |
| MMP-12 | 282.1 (196.6-352.8) | 289.4 (217.5-382.6) | 1.08 (0.96-1.22) | 0.775 |
| OLR-1 | 217.7 (122.2-404.5) | 200.8 (121.5-382.6) | 0.93 (0.77-1.14) | 0.839 |
|  |  |  |  |  |
|  | **Cases (N=478)^a^** | | **Low versus high**  **alveolar bone loss** | |
|  | *N=117* | *N=115* |  |  |
|  |  |  |  | |
| *Protein (pg/mL)* | *Low alveolar bone loss*  *median (IQR)* | *High alveolar bone loss*  *median (IQR)* | *Exp(B)*  *95 % CI* | *Adjusted*  *P-value* |
|  |  |  |  |  |
| OSM | 6.9 (4.6-10.3) | 6.9 (4.8-12.0) | 1.08 (0.89-1.31) | 0.839 |
| TGFA | 15.4 (11.3-21.6) | 15.4 (9.5-22.5) | 0.98 (0.85-1.12) | 0.878 |
| TNF | 16.2 (14.2-19.2) | 16.8 (13.6-19.7) | 1.04 (0.97-1.13) | 0.775 |
| TNFSF-10 | 454.9 (393.0-559.9) | 502.5 (432.8-586.5) | 1.07 (1.00-1.14) | 0.730 |
| TNFSF-12 | 687.2 (593.3-836.5) | 694.9 (580.7-795.0) | 0.99 (0.93-1.05) | 0.854 |
| TSLP | 0.1 (0.0-0.1) | 0.1 (0.0-0.1) | 0.85 (0.59-1.21) | 0.783 |
| VEGFA | 545.1 (390.6-760.8) | 607.1 (383.7-938.0) | 1.10 (0.95-1.28) | 0.775 |
|  |  |  |  |  |

Abbreviations: *Exp(B)* exponential regression coefficient, *95% CI* confidence interval

*P*-value based on linear regression model with log protein level as the dependent variable. The model was adjusted for the effects of age, gender and ever smoked.

a) 4 participants excluded from analyses due to missing serum data.

b) hs-CRP measured in mg/L

**Appendix Table 5.** Serum concentrations of EGF, OLR-1, MMP-12 and hs-CRP in cases and controls stratified by 10 year age groups.

|  |  |  |  |  |  |
| --- | --- | --- | --- | --- | --- |
| **Serum level in pg/mL** | **Control**  **Median (95% CI)** | **Case**  **Median (95% CI)** | **Exp(B)^a^**  **(95% CI)** | ***P*-value^a^** | **AUC**  **(95% CI)** |
|  |  |  |  |  |  |
| EGF |  |  |  |  |  |
| *30-40 years* | 550.4 (261.6-678.9) | 132.4 (70.6-199.9) | 0.34 (0.24-0.49) | < 0.001 | 0.82 (0.73-0.90) |
| *41-50 years* | 596.3 (277.2-763.7) | 126.3 (46.0-304.2) | 0.28 (0.22-0.38) | < 0.001 | 0.76 (0.69-0.83) |
| *51-60 years* | 573.4 (325.5-742.9) | 116.1 (46.9-422.6) | 0.21 (0.15-0.30) | < 0.001 | 0.77 (0.71-0.83) |
| *61-70 years* | 482.0 (313.6-664.5) | 102.3 (49.2-253.1) | 0.24 (0.14-0.42) | < 0.001 | 0.78 (0.70-0.86) |
|  |  |  |  |  |  |
| OLR-1 |  |  |  |  |  |
| *30-40 years* | 388.1 (249.2-546.7) | 255.7 (154.9-369.5) | 0.70 (0.54-0.90) | 0.005 | 0.69 (0.59-0.79) |
| *41-50 years* | 412.9 (264.4-602.7) | 219.8 (137.3-347.9) | 0.61 (0.51-0.72) | < 0.001 | 0.70 (0.64-0.77) |
| *51-60 years* | 378.6 (230.4-546.9) | 192.1 (119.1-377.2) | 0.59 (0.47-0.73) | < 0.001 | 0.69 (0.62-0.76) |
| *61-70 years* | 349.4 (235.9-514.6) | 212.3 (119.1-358.3) | 0.58 (0.41-0.83) | 0.002 | 0.69 (0.59-0.79) |
|  |  |  |  |  |  |
| MMP-12 |  |  |  |  |  |
| *30-40 years* | 178.9 (147.4-228.0) | 196.4 (132.7-261.5) | 0.99 (0.83-1.2) | 0.923 | 0.54 (0.42-0.67) |
| *41-50 years* | 171.2 (140.1-208.5) | 232.5 (178.2-291.1) | 1.36 (1.22-1.53) | < 0.001 | 0.73 (0.67-0.79) |
| *51-60 years* | 224.1 (171.8-289.5) | 290.1 (217.5-358.4) | 1.25 (1.1-1.4) | < 0.001 | 0.67 (0.60-0.74) |
| *61-70 years* | 196.2 (177.7-277.5) | 317.9 (241.9-424.9) | 1.57 (1.30 (1.90) | < 0.001 | 0.78 (0.70-0.87) |
|  |  |  |  |  |  |
| hs-CRP^b^ |  |  |  |  |  |
| *30-40 years* | 1.8 (0.9-3.7) | 1.9 (0.6-8.4) | 1.09 (0.68-1.74) | 0.725 | 0.51 (0.37-0.64) |
| *41-50 years* | 1.7 (0.8-3.6) | 2.4 (1.3-5.4) | 1.39 (1.03-1.88) | 0.029 | 0.61 (0.54-0.67) |
| *51-60 years* | 1.6 (0.8-2.9) | 3.4 (1.8-6.6) | 2.1 (1.6-2.7) | < 0.001 | 0.71 (0.64-0.78) |
| *61-70 years* | 1.5 (0.8-4.0) | 3.3 (1.8-6.9) | 1.79 (1.18-2.71) | 0.006 | 0.71 (0.60-0.83) |
|  |  |  |  |  |  |

a) *P*-value based on linear regression model with log protein level as the dependent variable. The model was adjusted for the effects of age, gender and ever smoked.

b) hs-CRP measured in mg/L

**References**

Holmlund A, Hedin M, Pussinen PJ, Lerner UH, Lind L. 2011. Porphyromonas gingivalis (pg) a possible link between impaired oral health and acute myocardial infarction. Int J Cardiol. 148(2):148-153.

Joss A, Adler R, Lang NP. 1994. Bleeding on probing. A parameter for monitoring periodontal conditions in clinical practice. J Clin Periodontol. 21(6):402-408.
